# High Sensitive and Selective Sensing of Hydrogen Peroxide Released from Pheochromocytoma Cells Based on Pt-Au Bimetallic Nanoparticles Electrodeposited on Reduced Graphene Sheets

**DOI:** 10.3390/s150202709

**Published:** 2015-01-26

**Authors:** Guangxia Yu, Weixiang Wu, Xiaoqi Pan, Qiang Zhao, Xiaoyun Wei, Qing Lu

**Affiliations:** Key Laboratory of Environment and Health, Ministry of Education & Ministry of Environmental Protection, and State Key Laboratory of Environmental Health (Incubating), School of Public Health, Tongji Medical College, Huazhong University of Science and Technology, Wuhan 430030, China; E-Mails: yu266021@163.com (G.Y.); sam_woo123@foxmail.com (W.W.); xiaoqipan0913@163.com (X.P.); farutong@163.com (Q.Z.); weixiaoyun2046@163.com (X.W.)

**Keywords:** hydrogen peroxide sensor, bimetallic nanoparticles, reduced graphene sheets, electrocatalysis, cancer cells, oxidative stress

## Abstract

In this study, a high sensitive and selective hydrogen peroxide (H_2_O_2_) sensor was successfully constructed with Pt-Au bimetallic nanoparticles (Pt-Au NPs)/reduced graphene sheets (rGSs) hybrid films. Various molar ratios of Au to Pt and different electrodeposition conditions were evaluated to control the morphology and electrocatalytic activity of the Pt-Au bimetallic nanoparticles. Upon optimal conditions, wide linear ranges from 1 µM to 1.78 mM and 1.78 mM to 16.8 mM were obtained, with a detection limit as low as 0.31 µM. Besides, due to the synergetic effects of the bimetallic NPs and rGSs, the amperometric H_2_O_2_ sensor could operate at a low potential of 0 V. Under this potential, not only common anodic interferences induced from ascorbic acid, uric acid and dopamine, but also the cathodic interference induced from endogenous O_2_ could be effectively avoided. Furthermore, with rat pheochromocytoma cells (PC 12) as model, the proposed sensor had been successfully used in the detection of H_2_O_2_ released from the cancer cells. This method with wide linear ranges and excellent selectivity can provide a promising alternative for H_2_O_2_ monitoring *in vivo* in the fields of physiology, pathology and diagnosis.

## 1. Introduction

H_2_O_2_ is an indicator of oxidative stress [1], which is closely related to the physiological and pathological events such as aging, cancer, ischemia/reperfusion injury, traumatic brain injury, impaired learning and memory functions, and so on [2,3]. In view of the importance of H_2_O_2_, development of real-time monitoring strategies for H_2_O_2_ *in vivo*, which can enable researchers to understand the chemical nature of oxidative stress in these physiological and pathological events, has aroused great attention in recent years. Several analytical technologies have been described for H_2_O_2_ determination in cellular environments, including spectrophotometry [4], electrochemical sensors [5], fluorimetry [6] and automatic potential titration [7]. Among these methods, amperometric techniques have attracted more and more attention in biological applications recently on account of their merits including excellent temporal resolution for measurements as well as great potential to image the dynamic release process of H_2_O_2_ [8]. Additionally, amperometric methods can also provide much more precise kinetic information on the exocytotic process itself and allow the efficiency evaluation of the whole process in real-time [9].

However, when employed for practical applications, H_2_O_2_ sensors are still up against primary challenges including high selectivity and wide linear range of the determination. On the one hand, within the biological environments, glucose, uric acid (UA), ascorbic acid (AA), dopamine (DA) as the anodic interferents and the endogenous oxygen as the cathodic interferent are usually co-existence with H_2_O_2_ [10]. Consequently, high selectivity is essential during the detection of H_2_O_2_ in the cellular environments. On the other hand, H_2_O_2_ holds a rather low concentration under normal physiological conditions while the concentration of H_2_O_2_ may increase to 10^−3^ mol under pathological conditions such as suffering ischemia/reperfusion injury or cancers [11]. Thus, it is necessary to conduct quantitative measurements for H_2_O_2_ with a broad linear range to figure out the role that H_2_O_2_ plays in physiological and pathological processes.

During the recent decades, bimetallic nanoparticles have been applied for the detection of H_2_O_2_ [12]. Compared with the corresponding monometal nanoparticles, bimetallic possesses higher catalysis, better resistance to deactivation, and greater selectivity [13]. Tsai *et al.* [14] fabricated electrochemical sensor based on the Au-Ag alloy nanoparticles, which exhibited high sensitivity and good selectivity for H_2_O_2_. Niu *et al.* [15] proposed novel snowflake-like Pt-Pd bimetallic clusters modified screenprinted gold nanofilm electrode for H_2_O_2_ and glucose sensing. In addition, bimetallic nanoparticles such as Pt/Ag and Pt/Cu [16], Pt/Pd [17], Pd/Cu [18], Rh/Pd [19] and Ru/Rh [20] were applied for constructing an electrochemical platform for determination of H_2_O_2_, and most of them possessed great potential.

Reportedly, platinum nanoparticles (Pt NPs) exhibit good electrocatalytic behaviors towards hydrogen peroxide and have been extensively used for H_2_O_2_ reduction at negative potentials [21], which can easily avoid the anodic interferents like AA, UA. However, Pt NPs modified electrodes could suffer from poor limit of detection and unstable baseline in most cases. Moreover, the cathodic interference of O_2_ is hard to be averted on account of its exceedingly negative reduction potential. Found from bimetal researches, the addition of a second metal, such as Au, could greatly improve the electrocatalytic and sensing performance of Pt, owing to the unique electrochemical properties of Au NPs as well as its ability to protect Pt from being poisoned by the reduction intermediates [22]. In the recent years, reduced graphene sheets have attracted a great deal of attention due to their high specific surface area, excellent electronic conductivity, and high-chemical stability [23], which can improve the electrocatalytic activity and provide more active site for the anchoring of substrate [24].

In this study, a novel H_2_O_2_ sensor based on Pt-Au/rGSs/GCE was fabricated by cyclic voltammetry method using the surfactant Brij 58 as a soft template. The results had indicated the addition of Au and rGSs could effectively improve the electrocatalytic properties and stability of Pt towards the reduction of H_2_O_2_. The proposed H_2_O_2_ sensor, possessing high sensitivity, excellent selectivity, good reproducibility and long-term stability, had been proven to be promising for the practical application in the real-time monitoring of H_2_O_2_ *in vivo*.

## 2. Experimental Section

### 2.1. Reagents

H_2_PtCl_6_·6H_2_O, HAuCl_4_·3H_2_O, Brij 58, catalase and lipopolysaccharide (LPS) were obtained from Sigma Aldrich (Shanghai, China). H_2_O_2_ (30%) was purchased from Sinopharm Chemical Reagent Co. Ltd (Shanghai, China). The reduced graphene sheets were obtained by the reduction of graphite oxide (obtained from Alfa Aesar, Ward Hill, MA, USA) via a chemistry synthetic route involving ultrasonic exfoliation and chemical reduction [25]. PC 12 cells were obtained from Chinese Academy of Sciences cell bank. All the other reagents were of analytical grade and double-distilled water was applied for all the aqueous solutions.

### 2.2. Apparatus

Electrochemical measurements were carried out employing a conventional three-electrode system, consisting a bare or modified glass carbon electrode with diameter 3 mm as the working electrode, a saturated calomel electrode (SCE) as the reference electrode and a platinum wire as the counter electrode. The cyclic voltammetry (CV) and current-time measurements were performed on a CHI830C electrochemical workstation (Shanghai Chenhua Apparatus, China). Field emission scanning electron microscopy (FESEM) and X-ray energy dispersive spectrometer (EDS) were taken at a Sirion 200 field-emission scanning electron microanalyzer with EDAX energy disperse spectroscopy (FEI, Netherlands). The morphology of the samples was characterized using high resolution transmission electron microscopy HRTEM (JEM-2100F, JEOL, Tokyo, Japan) equipped with selected area electron diffraction (SAED). The phase structures of the samples were determined by X-ray diffraction (XRD) (X'Pert Pro; Cu Kα, λ = 0.1542 nm, Philips, Amsterdam, The Netherlands).

### 2.3. Preparation of Pt-Au/rGSs Modified Electrode

GCE was polished with 0.05 µm alumina slurry, and then sonicated successively in nitric acid (1:1), ethanol and twice-distilled water. After that, 5 µL of 1 mg·mL^−1^ rGSs suspension was pipetted onto the GCE surface and dried under an infrared lamp to obtain rGSs/GCE. Thin Pt-Au alloy film was deposited on rGSs/GCE by cyclic voltammetry method in the aqueous solution of 2 mM H_2_PtCl_6_·6H_2_O and 1 mM HAuCl_4_·3H_2_O with 1.0 wt% Brij 58 under the potential from 0.4 V to −0.5 V at the scan rate of 100 mV·s^−1^ for five cycles. After the deposition, the film was rinsed with double-distilled water and then dried at room temperature for further use.

### 2.4. Detection of H_2_O_2_ Released from PC 12 Cells

The PC 12 cells were maintained in Roswell Park Memorial Institute (RPMI-1640) medium containing 10% fetal bovine serum, 100 unit·mL^−1^ penicillin, and 100 μg·mL^−1^ streptomycin. In the proliferative period, cells were cultured at 37 °C with 5% CO_2_ for about 24 h. Then, the cells were centrifuged to obtain a cell-packed pellet (1.0−2.0 × 10^5^ cells·cm^−2^) for the electrochemical experiments. Cell counting was carried out under a microscope. And real sample measurements were performed in PBS containing 100 mM glucose.

## 3. Results and Discussion

### 3.1. Characterizations of the Pt-Au/rGSs Hybrid Films

**Figure 1.**
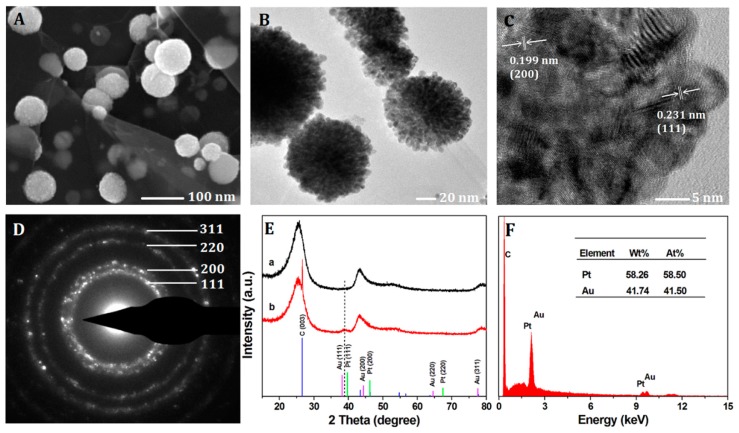
(**A**) FESEM image; (**B**) TEM image; (**C**) HRTEM image and (**D**) the corresponding SAED pattern of the Pt-Au/rGSs; (**E**) XRD patterns of (a) blank glassy carbon slice and (b) Pt-Au/rGSs modified on the glassy carbon slice; (**F**) EDS spectrum of the Pt-Au/rGSs.

The morphology and the structure of the Pt-Au/rGSs hybrid films were characterized by the FESEM and TEM measurements. As shown in Figure 1A, the typical FESEM image of Pt-Au/rGSs hybrid films and the as-prepared Pt-Au NPs with approximately 100 nm in diameter were found to be well dispersed on the graphene sheets. In these hybrid films, rGSs could support large surface areas for the deposition of nanoparticles. Thus, nanoparticles could be highly dispersed through the deposition on the both sides of these rGSs supports which could significantly improve the catalytic activity and sensors sensitivity as reported [26,27]. From the TEM image in Figure 1B, fluffy texture and irregular mesopores could be directly observed on the external surface of the Pt-Au particles, which might facilitate the charge-transfer and mass-transfer processes.

In addition, the HRTEM, SADE and XRD were employed to further determine the crystalline phase and chemical composition of the Pt-Au NPs. In Figure 1C, the HRTEM image revealed the lattice fringes of the NPs with interplanar spacing of 0.231 nm and 0.199 nm were in consistent with the (111) and (200) planes of Pt-Au alloy. In addition, the corresponding selected area electron diffraction (SAED) pattern (Figure 1D) indicated the polycrystalline nature of the NPs, and the diffraction rings could be readily indexed to the (111), (200), (220) and (311) planes of the Pt-Au alloy phases, demonstrating a face-centered cubic (fcc) structure. Furthermore, as shown in Figure 1E, XRD patterns of (a) bare glassy carbon slices (GCS) and (b) Pt-Au/rGSs modified on the GCS were recorded in the 2*θ* range of 15–80°. Several peaks were observed in the patterns of bare GCS which were due to the C element contained in GCS. In the case of Pt-Au/rGSs, two new peaks were observed. The peaks at 26.6° arose from the (003) plane of graphite in the rGSs. Besides, as shown by the dashed line, the (111) peak of Pt-Au/rGSs (39.03°) located between that of Pt (39.76°) and Au (38.18°) could further confirm the formation of the Pt-Au alloy. The elemental composition in Pt-Au/rGSs was analyzed via EDS (Figure 1F). The atomic number ratio of Au to Pt in the NPs film was 41.5:58.5, which was larger than the ratio in the precursor (with gold to platinum mole ratio of 1:2). The phenomenon could be attributed to the higher growth speed of the Au species than the Pt species under the provided electrodeposition conditions [28].

### 3.2. Optimization Parameters of the Pt-Au NPs Electrodeposition

**Figure 2.**
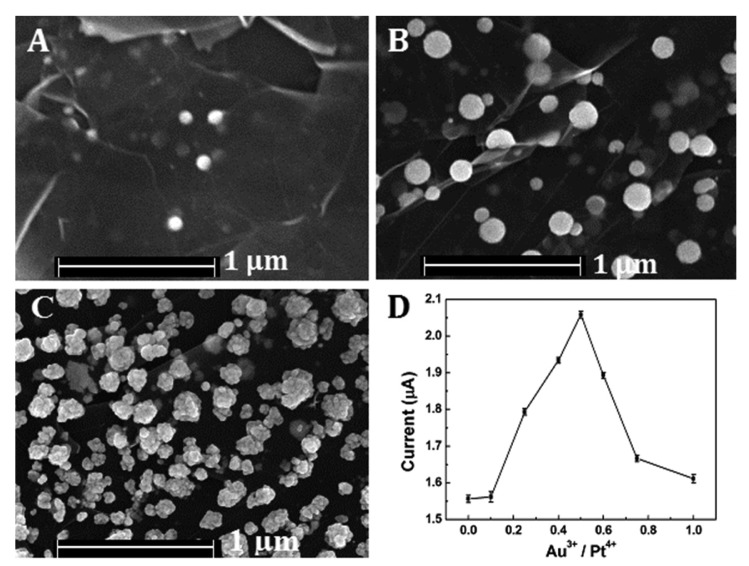
FESEM images of Pt-Au/rGSs hybrid films with different compositional ratios: Au^3+^/Pt^4+^ = (**A**) 0:1, (**B**) 1:2 and (**C**) 1:0; (**D**) plots of the peak current for the Pt-Au/rGSs/GCE in the presence of 0.1 mM H_2_O_2_ *vs.* different Au^3+^, Pt^4+^ compositional ratios.

Various molar ratios of Au to Pt (Au^3+^/Pt^4+^ = 0, 0:1, 1:4, 1:2, 1:1 and 1:0) had been employed for the preparation of Pt-Au alloy film in the study. The FESEM images of Pt-Au alloy film were shown in Figure 2. When there were only Pt species dissolved in the solution, very little NPs were deposited on the reduced graphene sheets. After the addition of Au species, much more uniform NPs were highly dispersed on the rGSs supports. However, when there were only Au species, as shown in Figure 2C, the NPs aggregated. The phenomenon could be attributed to the fact that the Au species, unlike Pt, tended to grow rapidly. The high-growth speed of Au species might be responsible for the NPs aggregating [28]. The effect of the ratio on the catalytic activity for H_2_O_2_ reduction was also evaluated. The results depicted in Figure 2D had indicated that the peak current of H_2_O_2_ increased significantly with the increase of Au^3+^ compositional ratio and achieved a maximum at the ratio up to 0.5 Then the current decreased as the compositional ratio further increased. The reason of the phenomenon might be that as the Au compositional ratio increased in the precursor solution, more homogeneous nanoparticles could be well distributed on the rGSs modified electrode, resulting in a larger surface area. Under this circumstance, the electrocatalytic activity of the alloy film to H_2_O_2_ was greatly improved. However, with the gradual increase of the Au ratio, the nanoparticles would aggregate, leading to the reduction of the catalytic active sites and the decrease of the electron transfer ability. Thus, 0.5 was chosen as the optimal Au compositional ratio for further study.

**Figure 3.**
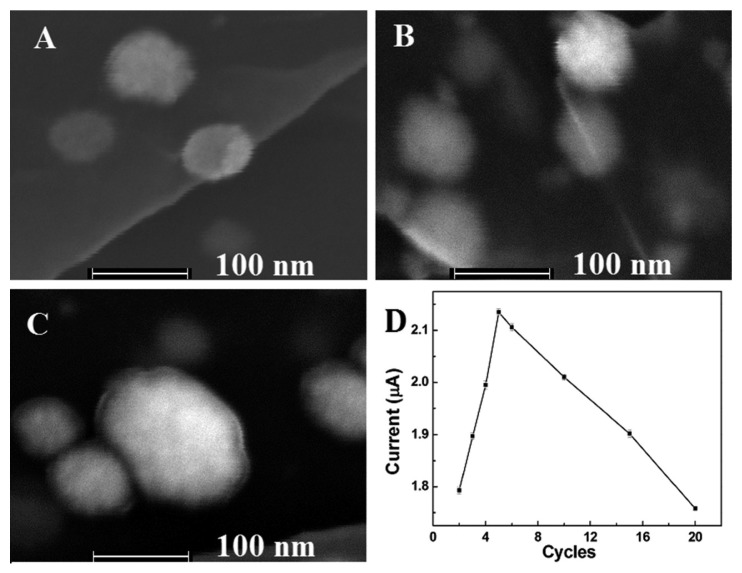
FESEM images of Pt-Au/rGSs hybrid films with different number of scan cycles: (**A**) 3 cycles; (**B**) 5 cycles and (**C**) 10 cycles, at the scan rate of 100 mV·s^−1^; (**D**) plots of the peak current for the Pt-Au/rGSs/GCE in the presence of 0.1 mM H_2_O_2_ *vs.* different number of electrodeposition scan cycles.

The effect of number of scan cycles during the CV deposition process on the size and morphology of Pt-Au nanoparticles were also discussed. The results had shown that the intensities of the nanoparticles increased with the increase of the number of scan cycles from 3 to 10 cycles. However, when the number of scan cycles increased to 10 cycles, nanoparticles with diameter lager than 20 nm were appeared, and the nanoparticles became non-uniform. (Figure 3A–C). Then the catalytic reduction to H_2_O_2_ was investigated. As shown in Figure 3D, the reduction peak current of H_2_O_2_ firstly increased from two to five cycles, and then decreased as the number of scan cycles further increased. The highest reduction peak current of H_2_O_2_ was obtained at five cycles. The phenomenon might be because that although higher intensities could provide more catalytic active sites, surface area might decrease on account of an exceedingly larger diameter of the nanoparticles. Thus, five cycles was adopted.

**Figure 4.**
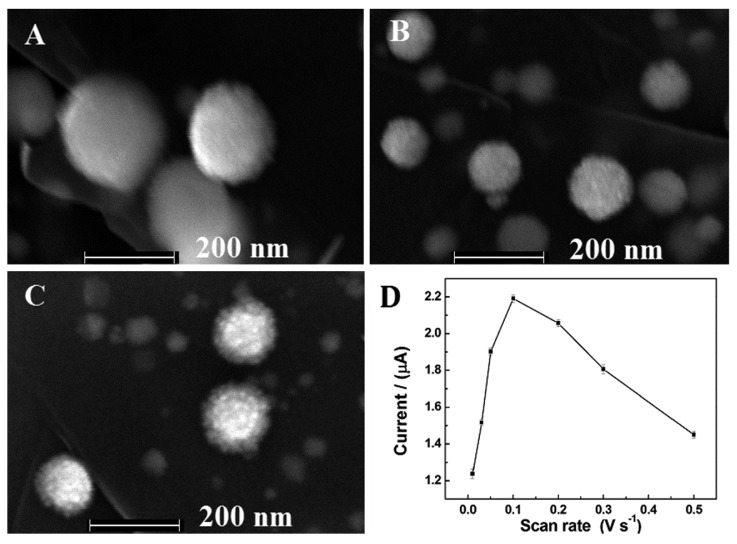
FESEM images of Pt-Au/rGSs hybrid films with different scan rates: (**A**) 50 mV·s^−1^; (**B**) 100 mV·s^−1^ and (**C**) 500 mV·s^−1^; (**D**) plots of the peak current for the Pt-Au/rGSs/GCE in the presence of 0.1 mM H_2_O_2_ *vs.* different electrodeposition scan rates.

In this experiment, the effects of scan rate were also evaluated. As the result shown in Figure 4, the homogeneous Pt-Au bimetallic NPs with high catalytic activity towards the reduction of H_2_O_2_ were obtained when the scan rate was 100 mV·s^−1^. Hence, the optimal scan rate was chosen as 100 mV·s^−1^.

### 3.3. Electrochemical Performances of the Pt-Au/rGSs/GCE towards H_2_O_2_

**Figure 5.**
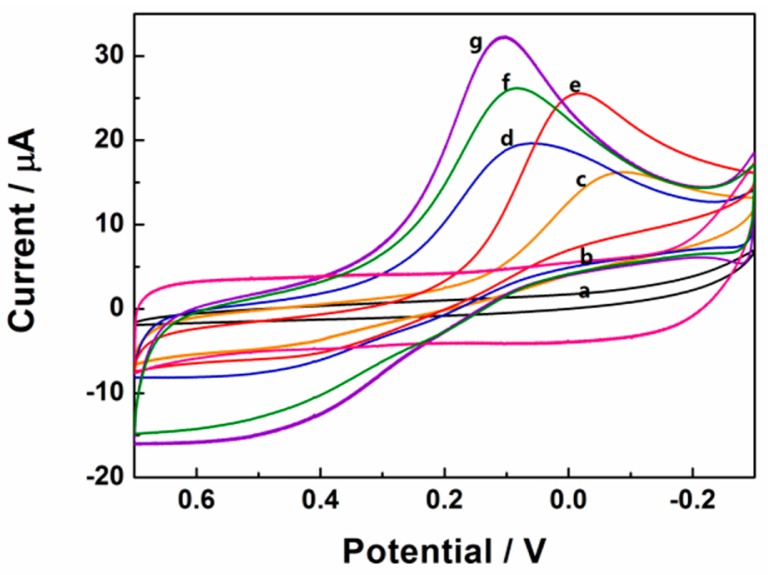
Cyclic voltammograms of (a) bare GCE; (b) rGSs/GCE; (c) Au/GCE; (d) Pt/GCE; (e) Pt-Au/GCE and (f) Pt/rGSs/GCE; (g) Pt-Au/rGSs/GCE in 0.1 M PBS (pH = 7.4) containing 0.1 mM H_2_O_2_ at a scan rate of 100 mV·s^−1^.

Cyclic voltammetry responses of 0.1 mM H_2_O_2_ in 0.1 M pH 7.4 PBS at different electrodes had been shown in Figure 5. After the addition of 0.1 mM H_2_O_2_, there was no observable reduction peak current on the bare and rGSs modified GCE, indicating that H_2_O_2_ could not be reduced at bare and rGSs modified GCE. In contrast, as shown in Figure 5, the reduction peak currents of 0.1 mM H_2_O_2_ on the differently modified electrodes increased as the following orders: Au/GCE < Pt/GCE < Pt/rGSs/GCE < Pt-Au/GCE < Pt-Au/rGSs/GCE. Larger current density and earlier reduction peak potential were obtained on the Pt-Au/rGSs/GCE than the other modified electrodes, confirming the excellent electrocatalytic activity of Pt-Au/rGSs/GCE towards H_2_O_2_ reduction. Besides, it was also worth recognizing that the reduction peak potential of Pt-Au/rGSs/GCE was between 0.3 V and 0 V, which was much higher than the previous reports. For instance, in the study of Meng, the potential range of the reduction of H_2_O_2_ was between 0 V and −0.4 V on the nanoporous gold modified GCE [29]. Similarly, the Pt-Au/G-CNT/GCE constructed by Lu's groups displayed a reduction peak current around −0.47 V [26]. The high reduction potential in this work suggested the Pt-Au/rGSs film had a high catalytic activity to H_2_O_2_ reduction. The excellent performance of Pt-Au/rGSs/GCE could be attributed to the tremendous improvement of the electrocatalytic and sensing performance of Pt in the presence of Au [30]. Besides, Pt-Au bimetallic NPs tended to be embedded in the rGSs homogeneously (Figure 2), which might help to facilitate the charge-transfer and mass-transfer processes. Therefore, Pt-Au/rGSs/GCE might have good performance in catalysis.

### 3.4. Amperometric Response and Calibration Curve for H_2_O_2_ Detection

**Figure 6.**
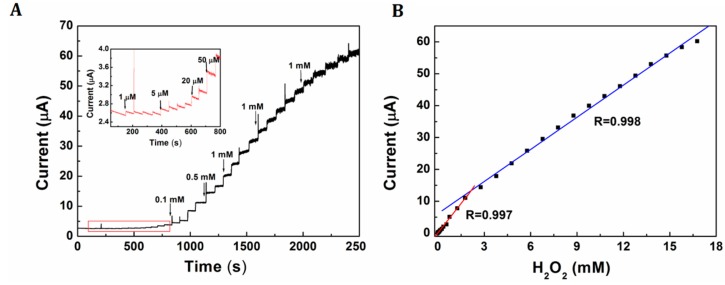
(**A**) Current-time response curve for successive injection of H_2_O_2_ at Pt-Au/rGSs/GCE measured at 0 V. Inset: Magnified image of the region in the rectangle; (**B**) Calibration curves of response current *vs.* H_2_O_2_ concentration at Pt-Au/rGSs/GCE.

Figure 6A shows a current-time curve of Pt-Au/rGSs/GCE with successive addition of H_2_O_2_ with different concentrations at an optimal potential of 0 V. The current increased linearly along with the addition of H_2_O_2_ in the range from 1 µM to 1.78 mM and 1.78 mM to 16.8 mM. Furthermore, the detection limit based on the signal to noise ratio (S/N) of 3 was calculated as low as 0.31 µM. The linear equations were *i* = −0.052 + 6.168 *C*; *i* = 6.125 + 3.359 *C* (*i* in μA, *C* in mM), with the correlation coefficient of 0.997 and 0.998 respectively (as shown in Figure 6B). The comparisons of analytical performance of the proposed sensor with other published H_2_O_2_ sensors had been displayed in Table 1. The results had revealed that the H_2_O_2_ sensor herein had comparative or even better advantages in lower detection limit and wider linearity range than several sensors reported recently. The higher sensitivity and wider linearity of this H_2_O_2_ sensor could be attributed to the addition of Au, which could effectively improve the electrocatalytic activity of Pt, especially the poisoning by adsorbed intermediates due to structure-induced promotional effects [22]. Furthermore, the addition of rGSs could increase the surface area and provide more active sites for the loading of H_2_O_2_.

### 3.5. Interference Study

**Figure 7.**
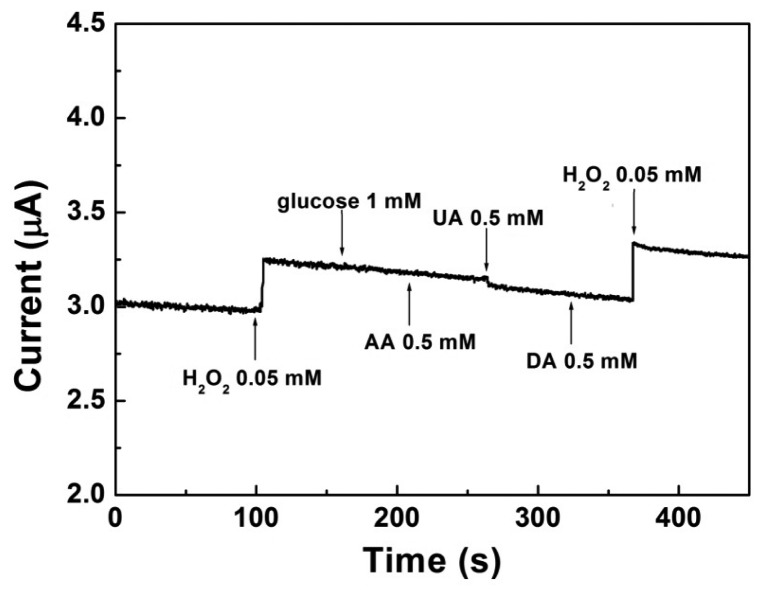
Current-time response curve at Pt-Au/rGSs/GCE for successive injection of H_2_O_2_, glucose, AA, UA and DA in 0.1 M PBS at 0 V.

The effect of common interfering electroactive substances such as glucose, AA, UA and DA were assessed and presented in Figure 7. It could be seen that an obvious amperometric response appeared when 0.05 mM H_2_O_2_ was injected at the first time. In the contrary, glucose (1 mM), AA (0.5 mM), UA (0.5 mM) and DA (0.5 mM) did not cause observable amperometric changes. When 0.05 mM H_2_O_2_ was added at the second time, the current changed proportionally even with the existence of the interferents, which indicated the proposed electrode had a superior selectivity to H_2_O_2_. As is well known, oxygen is another important interferent during the reduction process of H_2_O_2_. Thus, in most of the published researches, supporting electrolyte usually need to be deoxygenated by bubbling nitrogen for at least 20 min prior to each experiment section [29]. In this work, the reduction potential of H_2_O_2_ on Pt-Au/rGSs/GCE was around 0 V, which effectively avoided the interference of oxygen. Under this circumstance, the whole experiment in this study had been carried out without removing oxygen. All of the above results had indicated that the proposed H_2_O_2_ sensor could be used *in vivo*.

### 3.6. Reproducibility and Stability

The reproducibility and stability of Pt-Au/rGSs/GCE were also investigated. The relative standard deviation (RSD) of the current signal for 0.1 mM H_2_O_2_ was less than 2.1% for six measurements at the same electrode. Besides, the RSD was less than 4.7% at six modified electrodes prepared at the same condition. The results suggested a good reproducibility of the proposed H_2_O_2_ sensor. After being stored in the refrigerator at 4 °C for two weeks, the modified electrode still remained 93.2% of its original response, indicating that the fabricated H_2_O_2_ sensor exhibited long-term stability.

### 3.7. Determination of H_2_O_2_ Released from PC 12 Cells

**Figure 8.**
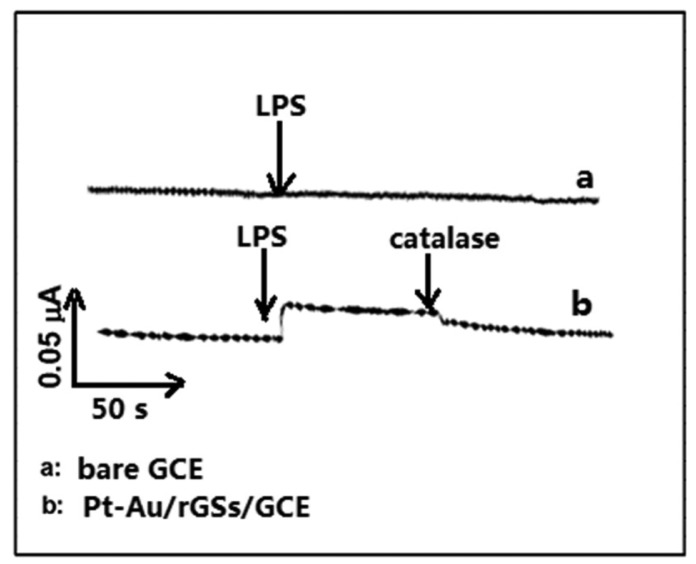
Amperometric responses at (a) bare GCE and (b) Pt-Au/rGSs/GCE in 0.1 M PBS containing PC 12 cells at the potential of 0 V with the addition of 5 µg·mL^−1^ LPS and 200 units·mL^−1^ catalase.

Figure 8 displays the amperometric responses at bare GCE and Pt-Au/rGSs/GCE in 0.1 M PBS containing PC 12 cells and 100 mM glucose. The increased cathodic current was observed at the Pt-Au/rGSs/GC electrode with the addition of 5 µg·mL^−1^ LPS, which was reported to induce H_2_O_2_ production from the cells [33].

Meanwhile, no response was attained at bare GCE with the same addition of LPS. After the injection of 200 unit·mL^−1^ catalase, the reduction current decreased at the Pt-Au/rGSs/GCE. The phenomena demonstrated that the reduction current at Pt-Au/rGSs/GC electrode was ascribed to the reduction of H_2_O_2_. These observations suggested the proposed electrode provided a promising alternative for the monitoring of H_2_O_2_ released from cells. Additionally, it could be potentially useful for further understanding of the role of oxidative stress in physiological and pathological processes.

## 4. Conclusions

In this study, Pt-Au bimetallic NPs were successfully synthesized on the rGSs modified GCE via a simple electrochemical deposition method. High dispersion and small particle size of the Pt-Au bimetallic NPs were achieved as respected by controlling the deposition conditions. Pt-Au/rGSs/GCE constructed upon optimal conditions exhibited high sensitivity and low-activation energy for the determination of H_2_O_2_. Wide linear ranges for the amperometric detection of H_2_O_2_ were respectively obtained from 1 µM to 1.78 mM and 1.78 mM to 16.8 mM, with a detection limit as low as 0.31 µM. Furthermore, the Pt-Au/rGSs/GCE showed high selectivity of H_2_O_2_ in the presence of common co-existing compounds like glucose, UA, AA, DA and oxygen. Good reproducibility and stability were also displayed on the proposed electrode. In view of all the unique properties, the fabricated sensor is promising for the practical application in the real-time detection of H_2_O_2_ *in vivo*, providing an alternative method to figure out the role that oxidative stress plays in physiological and pathological processes.

## Figures and Tables

**Table 1.** Comparisons of analytical performance of various H_2_O_2_ sensors.

**Types of Electrode**	**Detection Potential (V)**	**Linear Range (µM)**	**Detection Limit (µM)**	**Reference**
PDDA/t-MWCNT-Pt/GCE	−0.1	0.001–8	0.27	[21]
Pt-Au/G-CNTs/GCE	−0.47	0.002–8.561	0.6	[26]
Pt-CNT/GCE	−0.1	0.005–25	1.5	[31]
Se/Pt/GCE	−0	0.01–15	3.1	[32]
Pt-Au/rGSs /GCE	-0	0.001–1.78; 1.78–16.8	0.31	This work
